# Erythrocyte Deformability and Na,K-ATPase Activity in Various Pathophysiological Situations and Their Protection by Selected Nutritional Antioxidants in Humans

**DOI:** 10.3390/ijms222111924

**Published:** 2021-11-03

**Authors:** Jana Radosinska, Norbert Vrbjar

**Affiliations:** 1 Institute of Physiology, Faculty of Medicine, Comenius University in Bratislava, Sasinkova 2, 811 08 Bratislava, Slovakia; 2 Centre of Experimental Medicine, Slovak Academy of Sciences, Institute for Heart Research, Dúbravská Cesta 9, 841 04 Bratislava, Slovakia; norbert.vrbjar@savba.sk

**Keywords:** erythrocytes, deformability, Na,K-ATPase, pathophysiology, antioxidants, vitamins, phenolic compounds, hemorheology, human studies, human RBC

## Abstract

The physicochemical and functional properties of erythrocytes are worsened in a variety of diseases. Erythrocyte deformability refers to their ability to adjust their shape according to external forces exerted against them in the circulation. It is influenced by the functionality of the Na,K-ATPase enzyme, which is localized in their membranes. The proposed review is focused on knowledge regarding changes in erythrocyte Na,K-ATPase activity, and their impact on erythrocyte deformability in various pathophysiological situations observed exclusively in human studies, as well as on the potential erytroprotective effects of selected natural nutritional antioxidants. A clear link between the erythrocyte properties and the parameters of oxidative stress was observed. The undesirable consequences of oxidative stress on erythrocyte quality and hemorheology could be at least partially prevented by intake of diverse antioxidants occurring naturally in foodstuffs. Despite intensive research concerning the effect of antioxidants, only a small number of investigations on erythrocyte properties in humans is available in databases. It is worth shifting attention from animal and in vitro experiments and focusing more on antioxidant administration in human studies in order to establish what type of antioxidant, in what concentration, and in which individuals it may provide a beneficial effect on the human organism, by protecting erythrocyte properties.

## 1. Introduction

In the human population, a variety of diseases are associated with abnormalities in physicochemical and functional properties of erythrocytes. One important property of erythrocytes is their ability to change their shape depending on external forces exerted against them in the circulation. This ability of erythrocytes, also referred to as deformability, represents one determinant of whole blood viscosity affecting thus the blood flow in macrocirculation. Simultaneously, this property is very important for effective delivery of oxygen to target tissues—as erythrocytes are supposed to repeatedly pass through narrow capillaries in the microcirculation. It has been shown that skin blood flow after transfusion of packed erythrocytes is strongly dependent on their deformability [1]. This study also demonstrated that increase in hematocrit value is lower after transfusion of more rigid erythrocytes. Thus, the deformability is critical for the survival of erythrocytes in circulation. Impaired erythrocyte deformability was documented in hypertensive subjects [2,3,4,5,6,7], as well as in patients suffering from diabetes mellitus [8,9]. Consequently, the focus of scientists and researchers oriented to changes in erythrocyte deformability seems to be significantly relevant in clinical settings.

One of the systems regulating erythrocyte deformability is the Na,K-ATPase localized in their membranes. It represents the enzyme responsible for transport of three sodium ions out of the cell, simultaneously with the transport of two potassium ions into the cell—both against the concentration gradient under the consumption of one ATP molecule. Therefore, Na,K-ATPase contributes to unequal concentration of ions across the plasma membrane. This is necessary for the maintenance of resting membrane potential in all living cells in the organism, and its recovery after excitation in case of nerve and muscle cells. In addition, this transport system is responsible for the generation of low intracellular concentration of sodium ions providing the energy in form of the electrochemical gradient for sodium-calcium ion exchange across the plasma membrane as well as for a cotransport of a variety of substances inside the cell, e.g., sodium-glucose cotransport in renal tubular cells or in enterocytes. When focusing on erythrocytes, Na,K-ATPase maintains the optimal intracellular concentrations of cations regulating thus their volume and water homeostasis and in this way the surface area-to-volume ratio and cytoplasmic rheology—both determinants of erythrocyte deformability [10]. Thus, a decrease in Na,K-ATPase activity leads to a decrease in erythrocyte deformability. Several diseases affecting different organs throughout the human body are accompanied with the alteration of Na,K-ATPase activity in erythrocytes. The activity of the Na,K-ATPase in erythrocytes of patients was more times suggested as a biomarker with potential prognostic value for estimating the severity of various diseases. It is also well known that the functionality of this enzyme decreases in oxidative stress conditions [11,12]. More specifically, the oxidative stress-induced modifications of the Na,K-ATPase enzyme include its glutathionylation, nitrosylation, phosphorylation and carbonylation that are resulting in the conformation change and in decrease in enzyme activity [11]. Thus, antioxidants have been proposed as substances beneficial for the protection of Na,K-ATPase functions as well as for the erythrocyte condition in general. Considering the proposed theoretical background, the aim of this review is to summarize the knowledge regarding the changes in erythrocyte Na,K-ATPase functionality and erythrocyte deformability in various pathophysiological situations observed exclusively in human studies, as well as potential erytroprotective effects of natural nutritional antioxidants.

## 2. Erythrocyte Na,K-ATPase and Deformability in Various Patho/Physiological Situations

### 2.1. Cardiovascular and Pulmonary Diseases

The activity of Na,K-ATPase in erythrocyte membranes of patients suffering from heart failure was lower by approximately 60% compared with controls [13]. This finding can be at least partially related to observed significant decrease in erythrocyte deformability in heart failure patients when compared with healthy controls [14]. In patients suffering from stable coronary artery disease, the activity of this enzyme negatively correlated with the severity of disease, and it was related to significantly higher lipid peroxidation and lower cholesterol content in the erythrocyte membranes [12,15]. The link between coronary risk and the erythrocyte deformability was found in the PIVUS study [16], and this relation was independent of traditional risk factors. An evident decrease in erythrocyte deformability was observed in acute myocardial infarction (MI) [17] as well as in patients who suffered MI in the previous 3 to 12 months [18], but not in MI survivors who had the ischemic attack 3 years ago [19]. In the chronic obstructive pulmonary disease (COPD), changes in the structure and function of erythrocytes—including decreased activity of the Na,K-ATPase and the imbalance of redox status—were observed [20]. In COPD patients, the disease exacerbation was accompanied by the decrease in erythrocyte deformability in comparison with controls, as well as with COPD patients who received treatment [21]. The worsening the erythrocyte deformability was also detected in condition of high blood pressure—for review see [10]. In adult patients suffering from essential hypertension, an increased intracellular Na^+^ concentration, lowered Na,K-ATPase activity together with higher peroxidation of lipids were documented in erythrocytes [22,23,24,25], and almost the same was shown in patients with prehypertension [26]. Similarly, in the samples from hypertensive children, the Na,K-ATPase enzyme was also significantly inhibited, as indicated by kinetic studies showing a decrease of V_max_ value presumably due to alterations of the antioxidant status [27].

### 2.2. Endocrine and Metabolic Disorders

Another widely spread disease in developed countries, diabetes mellitus is also accompanied by alterations of erythrocyte properties including the decrease in Na,K-ATPase activity. A comparative study of 81 adult patients with type I diabetes mellitus, 87 patients with type II diabetes mellitus and 75 control healthy subjects showed lower enzyme activity in the type I diabetic patients, while type II diabetic patients did not differ from Na,K-ATPase activity of healthy subjects [28]. On the other hand, in a study oriented solely toward type II diabetes mellitus, increased hyperglycemia was followed by glycation of membrane enzymes, which together with lipid peroxidation induced a decrease in Na,K-ATPase activity and other changes in erythrocyte membrane [29]. The close relationship between the oxidative status and Na,K-ATPase activity of erythrocytes was also documented by a study with administration of antidiabetic agent metformin to patients suffering from type II diabetes mellitus [30]. Erythrocytes from patients with metabolic syndrome had higher concentration of cholesterol in the membrane, lower membrane fluidity as consequence of oxidative stress together with lower activity of Na,K-ATPase by 26% in comparison with the control group [31]. Additionally, the acute hyperglycemia induced in vitro by high concentration of glucose was associated with the reduced deformability of erythrocytes from healthy volunteers [32].

In patients with an abnormal thyroid function, the presence of Na,K-ATPase was significantly lower in erythrocytes from hyperthyroid patients while hypothyroid individuals showed the increased presence of this enzyme [33,34]. These observations are in agreement with a finding of lower erythrocyte deformability in newborns with congenital hypothyroidism [35]. Another study indicated an impairment of erythrocyte deformability in subclinical hypothyroidism that positively correlated with carotid intima-media thickness [36]. One may conclude that the impaired erythrocyte properties could negatively affect hemorheology in hypothyroid patients, thereby accelerating the progression of atherosclerosis.

### 2.3. Oncological Diseases

Several types of cancer also induced alterations in erythrocyte properties and the Na,K-ATPase enzyme functionality. In cervical cancer patients, the erythrocytes showed increased lipid peroxidation, increased fragility and lowered activity of Na,K-ATPase [37]. In breast cancer patients, the enzyme activity was lower before and also after the radiotherapy compared with healthy control subjects [38]. Patients diagnosed with oral cancer also showed lower activity of Na,K-ATPase in erythrocytes in advance of any treatment [39]. On the other hand, in patients suffering from head and neck squamous cell carcinoma, the oxidative stress generated by this disease induced a rearrangement of the lipid composition in erythrocyte membrane, followed by increased activity of Na,K-ATPase [40]. It has been discussed as a direct kinetic adaptation to consequences of membrane lipid peroxidation, as the erythrocytes of these patients were more permeable, leading to an ionic imbalance and promoting the hypoosmotic fragility. Therefore, in an attempt to counteract passive ions leaks, the enzyme activity increased. The Na,K-ATPase activity was also higher in erythrocytes of patients with non-small-cell lung cancer and glioblastoma multiforme in comparison with healthy controls [41]. Noteworthy, Na,K-ATPase activity in erythrocyte membranes decreased after the combined chemoradiotherapy, but did not reach the level observed in the control group.

Regarding erythrocyte deformability, it was found to be significantly lower in patients with differentiated thyroid cancers than in healthy individuals and was not modified after radioiodine treatment. Interestingly, the modification of oxidant–antioxidant stress values was not observable post-treatment, as well [42]. Comparing normal controls and multiple myeloma patients, a significant decrease in erythrocyte deformability was observed in cancer patients [43]. Taken together, from an erythrocyte perspective, various oncological diseases are associated with the impairment of erythrocyte Na,K-ATPase activity and deformability that could deteriorate the hemorheology in cancer patients affecting thus the oxygen delivery to tissues.

### 2.4. Neurological Diseases

The properties of erythrocytes were also altered in a variety of neuropsychiatric disorders. In children with autism, the oxidative stress was accompanied with lowered activity of the Na,K-ATPase [44,45], as well as with lowered erythrocyte deformability when compared with neurotypical children [46]. In patients suffering from epilepsy, the presence of Na,K-ATPase was significantly lower as documented by the decrease of specific binding of ouabain in the epilepsy group when compared with controls [47]. Studies oriented to Alzheimer disease showed an increased production of TBARS together with the increased activity of Na,K-ATPase in erythrocytes [48,49]. However, another study revealed lowered erythrocyte deformability with accompanied additional hemorheological abnormalities in patients suffering from Alzheimer’s disease when compared with control subjects [50]. In patients suffering from Parkinson’s disease, a significant reduction in the expression of Na,K-ATPase in erythrocytes was correlated with the significance of clinical features such as bradykinesia [51].

### 2.5. Renal Diseases

With respect to renal disease, the decreased activity of Na,K-ATPase was observed in acute as well as in the chronic renal failure but not in patients after kidney transplantation [52]. In the chronic phase of the disease, the enzyme showed worsened binding properties for the energy substrate ATP, as indicated by increased value of K_m_ [53]. These results are consistent with the observation of changes in erythrocyte shape and decreased deformability in patients with kidney disease that were independent of hemodialysis or diabetes mellitus conditions [54]. The erythrocyte deformation index was significantly improved following the living-donor renal transplantation [55], but not in conditions of peritoneal dialysis [56]. Interestingly, erythrocyte deformability index has been suggested as a sensitive marker for screening of diabetic nephropathy [57].

### 2.6. Red Blood Cell Disorders

In thalassemia—a congenital hemoglobinopathy associated with erythrocyte anomalies—impairments of erythrocyte deformability and functionality of Na,K-ATPase were shown. In a study of 38 patients with alpha-thalassemia and 48 patients with beta-thalassemia, the data suggested that reduced deformability of erythrocytes represents a major determinant of anemia in thalassemia [58]. Another study of 182 individuals suffering from alpha-thalassemia also documented the decrease in deformability of erythrocytes in comparison with 50 healthy volunteers. In addition, a tight connection between the severity of thalassemia and reduced antioxidant capacity in plasma was shown. The plasma levels of vitamin A, C, and E were significantly lower in alpha-thalassemic patients than in controls [59]. The above-mentioned abnormalities seem to be connected to the seriousness of thalassemia as in heterozygous sickle cell trait carriers, the erythrocyte deformability was similar like in control volunteers [60]. The properties of Na,K-ATPase in erythrocytes are also altered in several types of hemolytic anemia. It was shown that this enzyme exhibited normal kinetic parameters in control volunteers and in patients heterozygous for various defect hemoglobins. On the contrary, patients with alpha- or beta-thalassemia, displayed an abnormal biphasic character of the enzyme kinetics. The biphasic character correlated with the imbalance in the globin chain synthesis [61]. An investigation of the group of patients with wide range of thalassemia severity resulted in the observation of reduced Na,K-ATPase activity in milder form of the disease—i.e., in thalassemia-like cells, whereas it increased in severe alpha-thalassemia and in beta-thalassemia cells [62]. It is just a hypothesis at this point that such an increase in Na,K-ATPase activity could represent a compensatory mechanism in more severe forms of the disease.

### 2.7. Other Pathological Conditions

In patients suffering from galactosemia, the reason of decreased activity of Na,K-ATPase in erythrocytes was ascribed to a lowered total antioxidant status and a higher presence of free radicals in the blood [63]. Oxidative stress occurring in patients with rheumatoid arthritis induced lipid peroxidation together with a decrease of the Na,K-ATPase activity in erythrocytes [64]. Study oriented to the effect of alcohol consumption showed a decreased membrane fluidity in erythrocytes together with decreased activity of the Na,K-ATPase in chronic alcoholics [65]. Increased lipid peroxidation products with decrease in erythrocyte Na,K-ATPase activity were also reported in coal miners as compared to normal subjects [66]. It seems that erythrocytes, and particularly Na,K-ATPase enzyme in their membranes, are quite sensitive to alterations in the oxidative status what may further lead to deteriorating the erythrocyte deformability with consequences for the quality of macro- and microcirculation. In addition, it may be suggested that investigations of erythrocyte data collected in living patients suffering from various diseases seem to be relevant and helpful in characterizing the disease progression or treatment efficiency.

### 2.8. Aging

In addition to alterations induced by various pathologies, the properties of erythrocytes can also be influenced by natural biological processes like aging. A study of 33 male and 31 female healthy volunteers in the age from 18 to 76 years showed a significant age-dependent decrease of Na,K-ATPase activity in erythrocyte membranes. This correlated with the decreased total plasma antioxidant capacity. The observed alterations were similar in males and females [67,68]. However, it has to be mentioned that data concerning aging are controversial. Some other studies have shown that in healthy individuals, the enzyme activity was not influenced by aging [48,49,69].

More consistent are findings pointing to the age-related decrease in erythrocyte deformability in humans. The lower deformability found in the elderly group (by 19.1% than that of the young group) was partially normalized after incubation of erythrocytes with the Ca-blocker verapamil, while the ATP content was not altered [70]. Another study revealed an excessive count of aged erythrocytes in the blood of old persons that was accompanied with a decrease in ADP/ATP ratio, a decrease in Na,K-ATPase activity and intracellular accumulation of Na^+^ and Ca^2+^ ions [71].

### 2.9. Blood Transfusion

The properties of erythrocytes are very important for the process of blood transfusion. It was shown that flow erythrocyte properties are especially sensitive to storage what may result in circulatory abnormalities. Even though the blood samples were protected by cold storage and gamma irradiation, the hemodynamic properties of erythrocytes including the deformability were impaired long before the expiration date [72,73]. It is also important to consider that the deformability of freshly collected erythrocytes is variable already on the day of donation and the aging curve of erythrocyte deformability varied significantly among donors [74]. An additional source of storage-induced complications resulting in damage of erythrocytes may be the high concentration of glucose in storage mediums ranging 40–111 mmol/L; for review, see [75]. The functionality of the Na,K-ATPase also decreased in erythrocytes with the storage time of prophylactically irradiated blood samples. The inhibition of the enzyme ranged from 12.6% after 7 days to 44–55% after 14 or 28 days respectively [76].

## 3. Protection of Erythrocyte Na,K-ATPase and Deformability by Nutritional Antioxidants

Aiming to protect erythrocytes against oxygen radical overload, the effects of various antioxidants were investigated. The present review is focused on natural antioxidants that can be present in food and beverages.

Natural antioxidants can be characterized as molecules that react with free radicals and convert them to more stable products. There is no general consensus regarding the classification of naturally occurring antioxidant compounds. They include minerals that can serve as cofactors of antioxidant enzymes, antioxidant vitamins and phenolic compounds. The proposed review further focuses on vitamins with antioxidant properties and a heterogeneous group of molecules containing phenol rings in the structure.

### 3.1. Vitamins

Among the natural antioxidants commonly present in nutrition, an important role is attributed to vitamin C and vitamin E. Water-soluble vitamin C and lipid-soluble vitamin E can prevent lipid peroxidation in a variety of pro-oxidative situations like hyperglycemia, hypertension, metabolic syndrome, inflammation, etc.

#### 3.1.1. Vitamin C in Healthy Individuals

The supplementation of vitamin C in a dose of 1000 mg per day for 3 weeks in young healthy volunteers was followed by beneficial effect on multiple erythrocyte parameters including the deformability together with stimulation of the Na,K-ATPase as a consequence of increased presence of active enzyme molecules in erythrocytes together with improved ability of Na,K-ATPase to bind sodium ions. It was hypothesized that the better erythrocyte functionality could be reflected in enhanced quality of macro- and microcirculation, as indicated by multiple hemorheological parameters [77].

#### 3.1.2. Vitamin C and E in Hypertension

In hypertensive patients, lower concentrations of vitamin C and vitamin E were documented in plasma, together with higher levels of malondialdehyde, indicating higher predisposition for development of atherosclerosis [78]. Supplementation of vitamin C (1 g/day) + E (400 IU/day) for 8 weeks in patients with essential hypertension resulted in improved oxidative status, increased activity of Na,K-ATPase in erythrocytes and lowering the systolic as well as diastolic blood pressure as compared with patients receiving placebo [79]. Another observation of Na,K-ATPase enzyme in erythrocytes showed its significantly lower activity almost by 50% in hypertensive patients compared to normotensive subjects. Increased oxidative stress induced by the incubation of erythrocytes with oxidizing agent tert-butyl hydroperoxide (t-BHP) was mitigated by vitamin C administration in a concentration dependent mode in erythrocytes from hypertensive patients and also from normotensive subjects [80]. In the samples prepared for blood transfusion by prophylactic irradiation, the addition of vitamin C prevented the irradiation-induced loss of Na,K-ATPase activity in erythrocyte membranes [76].

#### 3.1.3. Vitamin E in Cancer Patients

The lipid-soluble vitamin E was proposed as a universal antioxidant and stabilizer of biological membranes [81]. Insufficiency of this vitamin was found in erythrocytes from patients suffering from cervical cancer. This study of erythrocytes showed markedly lower levels of vitamin E in membranes with concomitant increase in lipid peroxidation, increased osmotic fragility and significantly lower activity of Na,K-ATPase in these patients when compared with normal subjects [37]. In patients suffering from oral cancer disease, the activity of Na,K-ATPase in erythrocytes was also significantly lower as compared with healthy subjects. The documented reduction in enzyme activity was accentuated by six weeks of radiotherapy using a dose of 6000 cGY in five fractions per week. In patients supplemented by vitamin E (400 IU per day for entire period of radiotherapy), the enzyme activity reached the level similar to healthy controls [39].

#### 3.1.4. Vitamin C and E—In Vitro Experiments

During in vitro experiments, an incubation of erythrocytes from healthy donors with 2 mmol/L t-BHP for 5 min resulted in significantly reduced Na,K-ATPase activity by 43%. This effect was partially prevented by vitamin E in a dose-dependent manner in a range of 50–200 nmol [82]. In experimental model of glucose-induced oxidative stress on erythrocytes from venous blood, their incubation with glucose at a concentration of 10 or 20 mmol/L for 24 h was followed by hemolysis reaching 70–80%. The loss of membrane integrity was accompanied with the decrease in Na,K-ATPase activity. Treatment with vitamin E at 18 μg/mL (normal serum level) markedly reduced the hemolysis of erythrocytes together with enhancement of Na,K-ATPase activity. It was suggested that vitamin E supplementation may prevent rheological impairment of erythrocytes and delay or inhibit development of hyperglycemia-induced complications [83]. In vitro study oriented toward the effectiveness of vitamin C and vitamin E against acetaminophen-induced oxidative damage of erythrocytes showed that vitamin E (1 mg/dL of erythrocyte suspension) was more effective than vitamin C at the same dose in protection of hemoglobin, superoxide dismutase and Na,K-ATPase against undesirable acetaminophen action [84].

### 3.2. Phenolic Compounds

Another important group of natural substances with antioxidant properties is represented by flavonoids. These phytochemicals occur in high amounts in grapes, berries, cherries, apples, citrus fruits, onions, tomatoes, red wine, tea, etc. Despite intensive studies concerning their effect, only few investigations focusing on properties of human erythrocytes are available in the literature. The available studies explored mostly animal models. Among them, significant attention is oriented to quercetin, indicating its therapeutic potential against diabetes, inflammation, cardiovascular diseases, Alzheimer’s disease, different cancers, etc.; for review, see [85]. Regarding the influence of nutritional polyphenols on human erythrocytes, mainly in vitro studies are available.

#### 3.2.1. Quercetin—In Vitro Experiments

Quercetin interacts directly with the Na,K-ATPase molecule as it was shown by investigation of erythrocytes in vitro from blood of healthy donors. Quercetin in the presence of 1.5 micromolar concentration increased the activity of this enzyme by 31% [86]. Another experiment showed that pre-incubation of erythrocytes with quercetin (90 mg/mL) significantly prevented H_2_O_2_-induced loss of erythrocyte deformability [87]. However, one more in vitro study indicated that effect of quercetin on Na,K-ATPase activity could be concentration-dependent. In erythrocytes of healthy subjects as well as of patients with diagnosed type II diabetes mellitus, Na,K-ATPase activity was inhibited in the concentration of quercetin ranging from 10^−6^ mol/L to 10^−3^ mol/L, but not in lower concentration, i.e., in 10^−8^ mol/L [88]. A discrepancy between the results regarding the quercetin action could be also caused by the different solvents used to prepare a stock solution of quercetin, as quercetin is not water soluble.

Human erythrocytes from healthy volunteers were able accumulate large amounts of quercetin already after 5 min of incubation achieving as much as 85% of the initial amount of the flavonoid. Therefore, it was possible to hypothesize that human erythrocytes play a crucial role in the distribution and bioavailability of circulating flavonoids [89]. In vitro studies of the rheological properties of blood from healthy individuals in the presence of quercetin resulted in protection of erythrocytes including their deformability and osmotic resistance against the damage induced by arsenic [90]. Comparison of lipid-soluble quercetin and water-soluble dihydroquercetin showed a higher protective effect of quercetin indicating a prevalent importance of the interaction of flavonoids directly with the erythrocyte membrane [91].

#### 3.2.2. Resveratrol

##### Resveratrol—In Vitro Experiments

Resveratrol, another phytochemical with antioxidant properties, present mainly in red berries and red wine, increased the Na,K-ATPase activity in erythrocytes from healthy volunteers in vitro in a concentration dependent manner. In the presence of 0.1 μmol/L concentration, the resveratrol was ineffective, but it increased the enzyme activity at higher concentrations (1 and 10 μmol/L) by 37% or 66% respectively. Concerning the time necessary for the reaction with the Na,K-ATPase molecule, resveratrol seemed to be effective within 15 min of incubation and its effect lasted till 3 h of incubation. The antioxidant properties of resveratrol were confirmed by its ability to neutralize the effect induced by oxidizing agent t-BHP at a concentration of 1 μmol/L. These results suggested that resveratrol is a potent modulator of Na,K-ATPase in human erythrocytes [92].

##### Resveratrol—Post Myocardial Infarction and Heart Failure Patients

A placebo-controlled clinical trial of 40 post-MI patients randomized into two groups also showed beneficial effect of resveratrol. In the group receiving 10 mg resveratrol capsule daily for 3 months, the flavonoid exerted multiple protective effects on the cardiovascular system in patients with coronary artery disease developing its beneficial effect in addition to the routine medical therapy used in the secondary prevention of MI. Three-month lasting application of resveratrol improved the flow-mediated vasodilation, increased the deformability of erythrocytes, inhibited the aggregation of platelets, decreased LDL cholesterol level and improved left ventricular diastolic function [93]. Therefore, resveratrol itself in the absence of alcohol seems to have ameliorating effect on the Na,K-ATPase and hemorheological properties of erythrocytes, securing thus their better passage through the microvascular circulation post-MI. On the other side, the erythrocyte deformability remained unchanged after resveratrol administration to patients suffering from heart failure. However, resveratrol significantly reduced erythrocyte aggregation in these individuals, which can positively influence microcirculation as well [94].

#### 3.2.3. Epigallocatechin Gallate

##### Epigallocatechin Gallate—In Vitro Experiments

Epigallocatechin gallate (EGCG), one of the green tea polyphenols, via inhibiting the lipid peroxidation protected the activity of Na,K-ATPase in erythrocytes from healthy subjects when they were subjected in vitro to oxidation by t-BHP [95]. A study of erythrocytes from normal healthy subjects differing in age, showed that the above-mentioned tea catechin (EGCG) decreased the oxidation of membrane lipids throughout the range of human age with the highest effectivity in the group of oldest individuals [96,97]. Concerning the ionic homeostasis in erythrocytes, EGCG also mitigated most effectively the changes induced by t-BHP on Na,K-ATPase enzyme in all age groups; however, the effect was again more pronounced in the oldest age group [69]. A different study of elevated free radical formation induced by an increase in oxygen partial pressure (using in vitro hyperbaric oxygen model for erythrocytes from healthy volunteers) showed that EGCG effectively ameliorated hemorheological abnormality and enhanced erythrocyte deformability [98].

A study of the protection of erythrocyte membranes in type II diabetic patients against the oxidative damage by variety of tea catechins revealed positive effect for all investigated substances with the highest efficiency for EGCG. It was hypothesized that a higher intake of catechin-rich food by diabetic patients may provide some protection against the development of long-term complications of diabetes [99].

#### 3.2.4. Ferulic Acid—In Vitro Experiments

Examination of preventive in vitro protection of naturally occurring antioxidants like vitamin C, vitamin E, ferulic acid, EGCG against reactive oxygen species showed that the strongest antioxidant activity against the hydroxyl radical (one of the commonly present oxygen derived radicals in the human body) exhibited the ferulic acid. This compound completely protected the human erythrocytes against the loss of deformability caused by hydroxyl radicals [100]. Studying the effect of ferulic acid in concentration 10 or 100 μmol/L on the Na,K-ATPase enzyme, in vitro model of hyperglycemia showed partial protection of its activity by 14.13% and 22.81% respectively in erythrocytes exposed to 45 mmol/L glucose [101].

#### 3.2.5. Curcumin—In Vitro Experiments

Another bioactive compound curcumin, consisting of 2 feruoyl molecules, induced biphasic concentration dependent effect on the Na,K-ATPase activity from human erythrocytes when incubated in vitro. Curcumin in the presence of lower concentrations (below 1 µmol/L) stimulated the enzyme activity, while in higher concentrations (exceeding 10 µmol/L), the enzyme was inhibited by curcumin. In silico studies indicated that curcumin probably interacts with the same amino acids like ATP in the catalytic site of the Na,K-ATPase [102]. On the other hand, pretreatment with curcumin ameliorated the deteriorated deformability of erythrocytes subjected to hydrogen peroxide overload. In the range of 1–20 µmol/L of curcumin, the protection depended on the increasing concentration of curcumin with the highest effect in the presence of 20 µmol/L of curcumin [103].

## 4. Conclusions

It should be taken into consideration that a majority of experiments focused on the effect of antioxidant administration on erythrocyte properties were performed on animal models, not in humans. In addition, regarding the human studies, a significant portion refer to in vitro observations. 

Nevertheless, the quality of erythrocytes could be affected in various pathologies, leading to a deterioration in the patient condition. The present study is focused on two erythrocyte parameters—deformability and Na,K-ATPase activity. In fact, only a part of the studies was dealing with both of them simultaneously, so it was not always possible to point out a clear causal relationship between erythrocyte deformability and Na,K-ATPase activity in erythrocyte membranes in the mentioned pathologies. In addition, Na,K-ATPase enzyme activity is not the only factor affecting the erythrocyte deformability.

In the vast majority of studies, a clear link was observed between erythrocyte properties and parameters of oxidative stress. The undesirable consequences of oxidative stress on erythrocyte quality and hemorheology could be at least partially prevented by an intake of diverse antioxidants occurring naturally in foodstuffs. However, there are indices that antioxidants may also be pro-oxidative under certain conditions or may induce undesirable effects on erythrocytes. Thus, it is necessary to shift attention from animal and in vitro experiments and focus more on antioxidant administration in human studies in order to establish what type of antioxidant, in what concentration and in which individuals it may provide a beneficial effect on the human organism, by protecting erythrocyte properties.
